# W44X mutation in the WWOX gene causes intractable seizures and developmental delay: a case report

**DOI:** 10.1186/s12881-016-0317-z

**Published:** 2016-08-05

**Authors:** Loai Elsaadany, Mahmoud El-Said, Rehab Ali, Hussein Kamel, Tawfeg Ben-Omran

**Affiliations:** 1 Department of Pediatric, Hamad Medical Corporation, Doha, State of Qatar; 2 Department of Pediatric, Pediatric Neurology, Hamad Medical Corporation, Doha, State of Qatar; 3 Department of Pediatric, Clinical and Metabolic Genetic, Hamad Medical Corporation, Doha, State of Qatar; 4 Department of Neuro-Radiology, Hamad Medical Corporation, Doha, State of Qatar; 5 Department of Pediatric, Clinical Genetics, Weill-Cornell Medical College-Qatar, Clinical and Metabolic Genetic, Hamad Medical Corporation, PO Box 3050, Doha, State of Qatar

**Keywords:** WWOX, W44X, Seizure, Encephalopathy, Developmental delay, Whole Exome Sequencing (WES)

## Abstract

**Background:**

WW domain containing oxidoreductase (WWOX) gene was cloned in 2000; alteration has been seen in many cancer cells. It acts as a tumor suppresser by blocking cell growth and causing apoptosis. WWOX protein showed different expression of mice brain and spinal cord, for which deletion causes seizure and early death.

**Case presentation:**

Clinical and molecular characteristics of a consanguineous family show a homozygous mutation of WWOX gene at specific bases, causing a debilitating syndrome characterized by growth retardation, intractable epilepsy, intellectual disability, and early death.

Using Whole Exome Sequencing (WES), a novel homozygous mutation in the WWOX gene is identified in a consanguineous Arab family from Qatar with two daughters who presented with intractable seizure and developmental delay.

**Conclusion:**

The study presents the importance of human WWOX gene for brain development and the association between gene mutation and epileptic encephalopathy. It also highlights the power of WES particularly in clinically challenging cases.

## Background

WWOX gene spans a common fragile site FRA16D [1] Alteration of this gene has been associated with tumorigenesis [2] The expression of WWOX protein in the developing brain and spinal cord in the embryo and newborn mice together with the histological findings are showing a convincing evidence of its role in neural development [3]. This study identifies two siblings with novel WWOX gene mutation who presented for evaluation of epileptic encephalopathy. It highlights that the importance of whole exome sequencing in identifying causal mutations in challenging medical conditions [4].

## Case presentation

### Patients

**Fig. 1:**
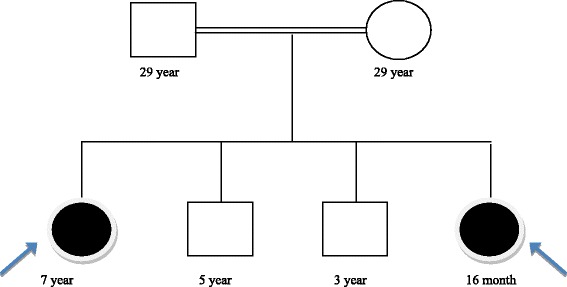
Family pedigree showing the consanguineous parents with affected sisters (black arrow) and unaffected brothers

The family came to medical attention because the eldest daughter presented with intractable seizure and developmental delay. A consanguineous family (Fig. 1a) with 4 children; 2 healthy boys, and the 2 affected girls. Informed consent was taken from the parents and obtained IRB (institutional review board) approval from the Ethics committee in the Medical Research Center at Hamad Medical Corporation.

### Patient 1

The proband is a 7-year-old girl, born at term after an uneventful pregnancy, admitted to the Neonatal Intensive Care Unit with transient tachypnea for one week. At 7 weeks of age, she was admitted to the hospital with abnormal movements. The abnormal movements described as unilateral alternating jerking of upper and lower limbs, which subsequently became bilateral. The movements were associated with eye gazing and blinking lasting for short periods and recurring up to 20–30 times per day. A video recording indicated typical myoclonic jerks. A trial of antiepileptic medications including phenobarbitone, clonazepam, phenytoin and levetiracetam, all failed to control the seizures. Developmentally, she had no eye contact and poor head control. Examination at 6 month of age, weight was 8.9 Kg (+1.43 SD), length 64 cm (+1.08 SD) and OFC 43 cm (+0.37SD). She had no dysmorphic features; generalized hypertonia. Eye examination showed both pupils were mid-dilated but reactive, however she was not blinking to strong light. There was no lens or corneal opacity. Fundoscopy showed bilateral optic discs’ pallor with normal retina. At 9 months of age, she had significant feeding difficulties necessitating nasogastric tube insertion initially, followed by gastrostomy tube feeding. At 2 years of age, she had progressive respiratory insufficiency requiring CPAP through tracheostomy, which eventually needed to be changed to mechanical ventilation. At the age of 4 years, there were poor head and trunk support with minimal limb movements, as well as progressive muscular weakness, hypertonia, and brisk deep tendon reflexes bilaterally. Currently, she is severely impaired with intractable seizure and in a vegetative state.

**Fig. 2:**
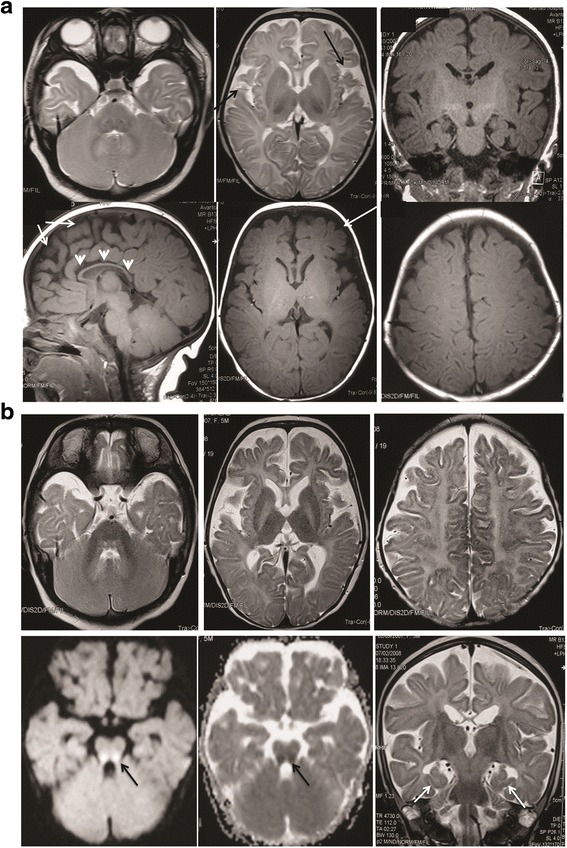
**a** Patient 1 initial MRI at age 9 weeks showing poor myelination with absent dark T2 and bright T1 signal in white mater tracts, marked widening of the Sylvian fissure (*black arrow*), moderate widening of the frontal sulci (*white arrow*) and mild ventricular dilatation in favor of frontal and temporal cortical atrophy. Marked symmetrical thinning of the corpus callosum (*arrow heads*). **b** Patient (1) follow up MRI at the age of 23 weeks showing significant progress in myelin loss with absent white mater myelination on T2 and marked progression in the frontal and temporal cortical atrophy. There is marked reduction of the size and deformity of the hippocampus (*black arrow*) with loss of its normal ram horn like whirled appearance. There is focal restricted diffusion along the cortico-spinal tract in the brain stem (*white arrow*)

Metabolic investigations including ammonia, lactate, plasma amino acids, urine organic acids, carnitine profile, plasma very long chain fatty acids, lysosomal enzymes studies, and CSF neurotransmitters, all were normal. Muscle and skin biopsies for mitochondrial studies were performed at another center and reported as normal. EEG showed frequent epileptic activity and abnormal background. Electroretinogram was normal, but visual evoked potential was delayed. Brain MRI showed progressive demyelination and atrophy of both frontal and temporal areas (Fig. 2a, b and c).

### Patient 2

The younger sister of the proband is a 20-month-old girl who was born after uneventful pregnancy and delivery. She presented with abnormal movement at the age of 7 weeks, in the form of focal tonic contraction on the right side with deviation of the mouth lasting from few seconds to 10 min associated with lip smacking and drooling. Antiepileptic medications including phenobarbitone, clobazam and topiramate showed partial response. The seizure attacks progressed for which she needed repeated hospital admissions. Developmentally, she could not roll, weak grasp for object and poor eye contact. Examination at 6 months of age, weight was 5.95 (−2.2 SD) length was 63.5 cm (−0.99 SD) and head circumference was 42 cm (0.16 SD). She had no dysmorphic features, no facial asymmetry. There was poor head control with increased tone and brisk reflexes in all limbs. At 8 months, she was admitted with acute bronchiolitis needing PICU admission for non-invasive ventilation; she had swallowing assessment for which she was started on nasogastric tube feeding. Currently, she is having severe psychomotor retardation and epileptic encephalopathy. Brain MRI showed progressive demyelination and atrophy of both frontal and temporal areas. (Fig. 2a, b).

At 16-month of age, she was admitted to PICU due to fever, acute respiratory failure and intractable Epilepsy. During this admission, she was seen by Gastroenterology team due to history of choking, gagging and aspiration. She had a G-tube and is currently entirely fed through a G-tube and nothing by mouth. In addition, she also seen by Orthopedics due to her healing right proximal humerus fracture and was noted to have mild neurogenic scoliosis. On exam, length was 85.1 cm, weight was 15.2 kg and head circumference was 48.5 cm. She had contractures of all 4 limbs. She was alert, but not interactive, sitting in a wheelchair. Strength was 2 in all extremities with normal muscle bulk and tone. Deep tendon reflexes were 2+ in the upper and lower extremities, and 3+ reflexes in the ankles with sustained clonus at the left and right. She had positive Babinski bilaterally.

She had plasma lactate, T4, TSH, AST, ALT, vitamin D, urine for organic acids, plasma and urine amino acids, CGH array, all were normal. EEG showed electro-clinical generalized tonic seizure, multiple myoclonic generalized seizures, multifocal spikes, generalized fast spikes, continuous generalized slowing, lack of normal sleep elements, and diffuse encephalopathy. A 24-h EEG showed 8 generalized spasms or tonic seizures per hour with multi-focal spikes while awake, and discontinuity of the background during sleep.

### Exome sequencing

Samples from the two patients were sent for WES and were done at GeneDx, MD USA (www.genedx.com), a commercial laboratory offering WES as a diagnostic service. Briefly, genomic DNA was extracted from whole blood. Exome sequencing was performed on exon targets isolated by capture using the Agilent SureSelect Human All Exon V4 (50 Mb) kit (Agilent Technologies, Santa Clara, CA) and was sequenced using the IlluminaHiSeq 2500 sequencing system with 100-bp paired-end reads (Illumina, San Diego, CA). The identified pathogenic variants were validated and were confirmed by Sanger sequencing. Whole-exome sequencing of patient 1 & 2 showed a novel homozygous W44X mutation [p.Trp44Stop (TGG > TAG): c.131 G > A in exon 2] in the *WWOX* gene. Segregation study showed both parents are heterozygous W44X mutation. The W44X pathogenic mutation in the WWOX gene is predicted to cause loss of normal protein function either through protein truncation or nonsense-mediated mRNA decay. This mutation was not observed in approximately 6000 individuals of European and African American ancestry in the NHLBI Exome Sequencing Project, indicating it is not a common benign variant in these populations.

## Discussion

WWOX gene is situated in the second most common fragile site of breakage known as FRA16D, which contains nine exons encodes a 414 amino acids protein [1] It contains two WW domains on the NH2 terminus of the protein and an SDR central domain with a chromosomal area characterized by a very high incidence of allelic loss and chromosomal rearrangements [1]. WWOX gene has been identified in many cancer cells, for example upregulation was found during prostatic and breast cancer progression from hyperplasia to metastasis [2] Targeted deletion of Wwox in mice has been found to cause growth retardation and early postnatal death [5]. Understanding the role of Wwox in the brain development showed that loss of function of Wwox gene causes seizure in rats [3] McDonald CB et al. [6] showed that the replacement of R25 and W44 within the WW1 domain with E66 and Y85, respectively, at corresponding structurally equivalent positions within the WW2 domain accounts for the lack of binding of the WW2 to PPXY (X is any amino acid) motifs within WBP1 (WW binding protein 1) and WBP2 (WW binding protein 2) adaptors. The Y residue of the PPXY WW-binding motif is a potential target of tyrosine phosphorylation. Indeed, tyrosine phosphorylation of this motif has been shown to regulate the interaction between several WW proteins and their interacting proteins [7]. These interactions could be a channel in understanding of the disease pathophysiology.

Autosomal recessive WWOX gene mutation has been identified in several families with a variable neurodevelopmental phenotypic spectrum [8–12]. In two families, early death with microcephaly, epileptic seizures, growth retardation and eye abnormalities (retinopathy and optic atrophy) result from homozygous early premature termination codons in WWOX gene [9, 10]. In two other families, cerebellar ataxia with epilepsy and mental retardation are consequences of missense mutation in the WWOX gene [10].

In 2015, Mignot et al. [11] reported seven families with WWOX-related encephalopathies and has concluded a genotype-phenotype correlation with variable phenotypic presentation. In four patients with two predicted null alleles, the clinical manifestations include severe psychomotor retardation, early intractable epilepsy, poor eye contact with retinal degeneration, secondary microcephaly, abnormal brain MRI and premature death. The other group of patients due to hypomorphic alleles showed a spinocerebellar ataxia type 12 phenotype that include cerebellar limb and gait ataxia, delayed psychomotor development, spasticity, hyporeflexia, and onset of seizures between 9 and 12 months of age. Our cases fit well with WOREE syndrome (WWOX-related epileptic encephalopathy). It was first described in four families in Mignot et al [11] of which three described as a WOREE syndrome having a similar presentation to the two families reported by Abdel-Salam et al. [8] and Ben Salem et al. [9] with early termination codons [8, 9, 11]. The most recent family reported had one case with a prenatal diagnosis of WOREE syndrome [12].

Epileptic encephalopathies have wide-range of disorders characterized by epilepsy with permanent detrimental effect on the developing brain [13]. Therefore, it is customary that patients with epileptic encephalopathy usually undergo a vast array of biochemical and molecular tests leading to an expensive workup. However, the diagnosis often remains elusive despite extensive testing which prolong the length of the diagnostic odyssey [14]. Hence, whole-exome sequencing is a powerful diagnostic tool that has been used recently to assist in the diagnosis of many monogenic neurometabolic disorders.

We have identified a consanguineous Qatari family with a novel W44X mutation in the WWOX gene. This mutation is predicted to cause loss of normal protein function either through protein truncation or nonsense-mediated mRNA decay. To the best of our knowledge there are ten families reported including our family, six of which are consanguineous Middle Eastern families [8–10, 12]. The other four families are non-consanguineous [11]. All reported cases presented with progressive severe epilepsy, psychomotor retardation, and brain abnormalities (Table 1).

**Table 1:** Clinical phenotypes in patients with Homozygous WWOX gene mutation

	Our study	Mylene valduga et al. [12]^a^	Ben Salem et al. [9]	Mignot et al. [11]	Abdel-Salam et al. [8]	Mallaret et al. [10]
No of Families	1	1	1	4	1	2
No of pts	2	2	1	5	1	4
Gender	2F	1F-1M	1M	4F-1M	F	3F-1M
Consanguinity	+	+	+	-	+	+
Ethnicity	Qatari	Turkish	Emirati	NA	Egyptian	Saudi-Palestinian
Acquired microcephaly	-	+	+	(3/5 - ) (2/5 +)^b^	+	NA
Neonatal hypotonia	-	-	+	+	+	NA
Psychomotor delay	+	+	+	+	+	+
Cerebellar ataxia	-	-	-	-	-	+
Spasticity	+	+	+	3/5 (-), 2/5 (+)	+	-
DTR	Exaggerated	Exaggerated	Exaggerated	3/5 Normal, 2/5 exaggerated^c^	Exaggerated	Diminished
Ophthalmological involvement	+	+	+	3+, 2-	+	+
Age of onset of epilepsy	2 month	3 month	2 weeks	Between <2m and 5m	Yes (2 month)	(9–12 m)
Seizure type	Myoclonic seizure	Infantile spasm	Infantile spasm	Focal and generalized tonic clonic and myoclonic	Focal and generalized tonic clonic and myoclonic	Generalized tonic-clonic
Response to antiepileptics	Partial	Partial	Yes	Partial	Partial	Yes (2/4), partial (2/4)
Brain MRI	Brain atrophy.	Brain atrophy.	Brain atrophy and polymicrogyria on the right frontoparietal region	Brain atrophy, 2 Normal^d^	Brain atrophy	Posterior white matter hyperintensities
Premature death	-	+	-	2 (+)-3(-)	+	-

*F* female, *M* male, *No* number, *pts* patients, *DTR* deep tendon reflexes, *NA* not applicable

^a^The case diagnosed prenatally was not included in the table as the comparison points are not applicable

^b^3/ 5 cases did not have microcephaly, while 2/5 cases have microcephaly

^c^2/5 spastic and 3/5 normal tone. The deep tendon reflex was normal in 3/5 while exaggerated in 2/5

^d^2 cases had normal MRI

## Conclusion

This family exemplifies the challenges in establishing an accurate diagnosis in patients presented with non-specific epileptic encephalopathy and we recommend performing WES as a first line test to reach the final diagnosis particularly in populations with high rates of consanguinity. Furthermore, the role of WWOX gene in brain development is crucial and the relationship between epileptic encephalopathy with gene mutation warrant further studies to determine the exact pathophysiology.

## Abbreviations

WES, Whole Exome Sequencing; WWOX, WW domain containing oxidoreductase; WOREE syndrome, WWOX-related epileptic encephalopathy
